# Long-term safety evaluation of mirtazapine: A real-world pharmacovigilance study based on the FAERS database

**DOI:** 10.1371/journal.pone.0340092

**Published:** 2026-03-06

**Authors:** Kaidi Zhao, Jiashu Liu

**Affiliations:** 1 Department of Dermatology, Xi’an Jiaotong University Second Affiliated Hospital, Xi’an, China; 2 Department of Dermatology, Xi’an Children’s Hospital, Xi’an, China; Northwestern University Feinberg School of Medicine, UNITED STATES OF AMERICA

## Abstract

**Background:**

Mirtazapine is widely used in the treatment of major depressive disorder (MDD), yet its real-world safety profile remains insufficiently evaluated.

**Methods:**

This study analyzed adverse event (AE, plural AEs) reports related to mirtazapine from the FDA Adverse Event Reporting System (FAERS) database between the first quarter of 2004 and the first quarter of 2025. Four disproportionality analysis methods were employed, including reporting odds ratio (ROR), proportional reporting ratio (PRR), Bayesian confidence propagation neural network (BCPNN), and multi-item gamma Poisson shrinker (MGPS). Additional analyses included sensitivity analysis, time-to-onset (TTO) evaluation, and Weibull distribution modeling.

**Results:**

A total of 17,953 reports were included, with females accounting for 54.1% and males for 37.7%. In 48.4% of the reports, patients were aged between 18 and 65 years. Overall, 65.0% of the reports were submitted by healthcare professionals. Known AEs such as somnolence (n = 887, ROR = 4.57, PRR = 4.52, EBGM = 4.50, IC = 2.17), QT interval prolongation (n = 259, ROR = 7.35, PRR = 7.32, EBGM = 7.27, IC = 2.86), suicidal ideation (n = 601, ROR = 6.65, PRR = 6.59, EBGM = 6.55, IC = 2.71), and rhabdomyolysis (n = 146, ROR = 3.66, PRR = 3.65, EBGM = 3.64, IC = 1.87) showed positive signals. In addition, several unexpected AEs also exhibited positive signals, including restless legs syndrome (n = 305, ROR = 17.05, PRR = 16.97, EBGM = 16.65, IC = 4.06), neuroleptic malignant syndrome (n = 158, ROR = 13.77, PRR = 13.74, EBGM = 13.53, IC = 3.76), and nightmare (n = 279, ROR = 8.02, PRR = 7.99, EBGM = 7.93, IC = 2.99). TTO analysis showed that 59.52% of AEs occurred within the first month of treatment. The results of the sensitivity analyses further supported the robustness of these findings.

**Conclusion:**

This study systematically assessed the long-term safety profile of mirtazapine using 21 years of real-world pharmacovigilance data from the FAERS database. The findings provide important evidence to support safe clinical use of mirtazapine and emphasize the need for continuous safety monitoring.

## Introduction

Major depressive disorder (MDD) is a common and disabling psychiatric condition that imposes a substantial burden on patients and global healthcare systems [1–3]. Antidepressants remain the cornerstone of MDD treatment, with various pharmacological classes developed to address its complex neurobiological mechanisms [4]. Among them, mirtazapine, a noradrenergic and specific serotonergic antidepressant (NaSSA), is widely used in clinical practice due to its unique dual mechanism of action [5]. It exerts its effects by antagonizing central α₂-adrenergic receptors as well as 5-HT₂ and 5-HT₃ receptors, resulting in distinct clinical benefits such as sedation and appetite stimulation, particularly favorable for patients with comorbid insomnia or appetite loss [6,7].

Although mirtazapine has demonstrated good efficacy and tolerability in clinical trials [8–10], these studies are often limited by small sample sizes, short durations, and strict inclusion criteria. As a result, rare, delayed-onset, or multifactorial AEs may not be adequately captured under controlled trial conditions. AEs commonly associated with mirtazapine include somnolence, weight gain, dizziness, and increased appetite. However, with its widespread use in clinical practice, several case reports have documented serious but less common AEs such as rhabdomyolysis [11,12] and somnambulism [13]. These findings underscore the importance of real-world pharmacovigilance studies to provide a more comprehensive and objective assessment of mirtazapine’s safety profile.

The U.S. Food and Drug Administration Adverse Event Reporting System (FAERS) is one of the largest publicly accessible spontaneous reporting databases worldwide and is extensively used for drug safety investigations in psychiatry, cardiology, dermatology, and other medical fields [14–16]. Despite the widespread use of mirtazapine, no large-scale, systematic safety evaluation based on FAERS data has been conducted to date. Therefore, this study aims to utilize the FAERS database, incorporating approximately 21 years of data, and apply multiple disproportionality analysis methods to identify potential AE signals associated with mirtazapine. The findings are expected to enrich the real-world safety evidence base of mirtazapine, enhance clinical awareness of potential risks, support rational prescribing decisions, and inform regulatory surveillance and risk communication.

## Methods

### Data source and data cleaning

**Fig 1:**
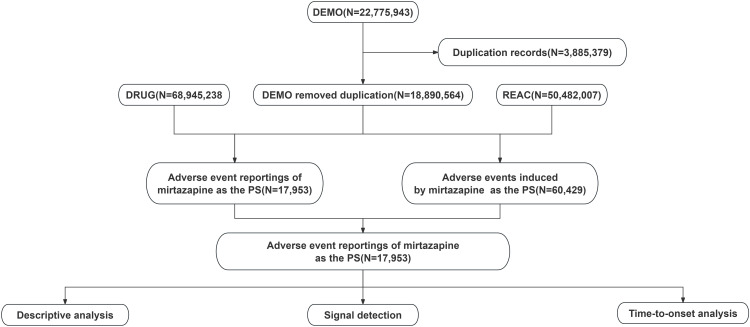
Flowchart illustrating the process of analyzing AEs associated with mirtazapine using the FDA Adverse Event Reporting System database. Abbreviations: DEMO, demographic information; DRUG, drug information; REAC, adverse event information; PS, primary suspected; AEs, adverse events.

The FAERS database collects spontaneously reported AE reports from physicians, nurses, pharmacists, patients, and other reporters. It is primarily used for post-marketing drug safety surveillance and the detection of potential safety signals. The FAERS database primarily consists of seven datasets: DEMO (Demographics), REAC (Reaction), DRUG (Drug), OUTC (Outcome), RPSR (Reporter), THER (Therapy), and INDI (Indication). In FAERS, the relationship between a drug and an AE report is categorized into four types including primary suspect (PS), secondary suspect (SS), concomitant (C) and interacting (I). We collected AE reports in which mirtazapine was identified as a PS drug between January 1, 2004 and March 31, 2025. Data were extracted on May 2, 2025. We extracted the following variables from these datasets: primary identifier (PRIMARYID), case identifier (CASEID), FDA receipt date (FDA_DT), therapy start date (START_DT), event date (EVENT_DT), age (AGE), sex (SEX), occupation code (OCCP_COD), reporter country (REPORTER_COUNTRY), role code (ROLE_COD), preferred term (PT), and system organ class (SOC). We performed deduplication in accordance with the FDA deduplication principles using three parameters: CASEID, FDA_DT, and PRIMARYID [17]. Duplicate reports were handled as follows: (1) when CASEID was identical, the report with the most recent FDA_DT was retained. (2) When both CASEID and FDA_DT were identical, the report with the highest PRIMARYID was retained. AEs were coded using the Medical Dictionary for Regulatory Activities (MedDRA, version 27.1) and classified at two levels: SOC and PT. The flowchart of this study is shown in Fig 1.

### Disproportionality analysis

To identify potential safety signals associated with mirtazapine, four commonly used disproportionality analysis algorithms were applied, including Reporting Odds Ratio (ROR), Proportional Reporting Ratio (PRR), Multi-item Gamma Poisson Shrinker (MGPS), and Bayesian Confidence Propagation Neural Network (BCPNN). These methods are based on 2 × 2 contingency tables to assess whether the reporting frequency of a specific AE for mirtazapine is statistically disproportionate compared to that of all other drugs in the database. A signal was considered statistically significant only if it met the threshold criteria of all four disproportionality analysis methods. The computational logic and specific formulas for these methods are presented in S1 and S2 Tables.

### Sensitivity analysis

In clinical practice, mirtazapine is often co-administered with other psychotropic agents such as escitalopram, sertraline, fluoxetine, venlafaxine, duloxetine, bupropion, and aripiprazole. To eliminate potential confounding effects from these concomitant medications, reports involving any of the above drugs were excluded, and a reanalysis was conducted.

### Time to onset (TTO) and Weibull Modeling

The TTO was determined by calculating the difference between the EVENT_DT field in the DEMO file and the START_DT field in the THER file. This interval represented the latency between the initiation of drug therapy and the onset of an AE. Records with missing, inconsistent, or implausible time values were excluded from subsequent analyses. To describe the temporal evolution of adverse event risk, a Weibull distribution model was fitted. This parametric approach allowed for the estimation of the hazard function over time, providing insights into whether the occurrence of AEs tended to increase, decrease, or remain constant during the exposure period.

All data processing and statistical analyses were performed using R software (version 4.3.0), primarily with the PhViD package (for the calculation of ROR, PRR, and IC), the openEBGM package (for EBGM), and ggplot2 (for data visualization).

## Results

### Baseline characteristics

A total of 17,953 reports were analyzed, with 9,711 (54.1%) involving female patients and 5,752 (37.7%) involving male patients. Most reports (48.4%) concerned individuals aged between 18 and 65 years. The top three reporting countries were the United Kingdom(27.3%), the United States(18.9%), and France(10.4%). Notably, 65.0% of the reports were submitted by healthcare professionals. Since the approval of mirtazapine, the annual number of related AE reports has increased year by year, reaching a peak in 2019, followed by a slight decline in recent years. Detailed baseline characteristics of the reports are presented in Table 1.

**Table 1:** Clinical characteristics of mirtazapine AE reports in the FAERS database (Q1 2004 - Q1 2025).

Characteristics	Numbers	Case proportion (%)
Number of reports	17953	
**Gender**		
Female	9711	54.1%
Male	5752	37.7%
Missing	1480	8.2%
**Age**		
<18	502	2.8%
18-65	8681	48.4%
＞65	4963	27.7%
Missing	3807	21.2%
**Top 10 Reported Countries**		
United Kingdom	4910	27.3%
United States	3394	18.9%
France	1852	10.4%
Germany	1600	8.9%
Italy	916	5.1%
Japan	703	3.9%
Sweden	589	3.3%
Canada	552	3.0%
Spain	393	2.2%
Netherlands	303	1.6%
**Reporter**		
Healthcare professional	11756	65.0%
Non-healthcare professional	4668	26.0%
Missing	1634	9.1%
**Reporting year**		
2004	313	1.7%
2005	354	2.0%
2006	344	1.9%
2007	406	2.3%
2008	386	2.2%
2009	445	2.5%
2010	550	3.1%
2011	713	4.0%
2012	570	3.2%
2013	644	3.6%
2014	871	4.9%
2015	1052	5.9%
2016	971	5.4%
2017	917	5.1%
2018	1447	8.1%
2019	1759	9.8%
2020	1585	8.8%
2021	1056	5.9%
2022	1035	5.8%
2023	1163	6.5%
2024	1105	6.2%
2025	267	1.5%

### Distribution of AEs across SOC

**Fig 2:**
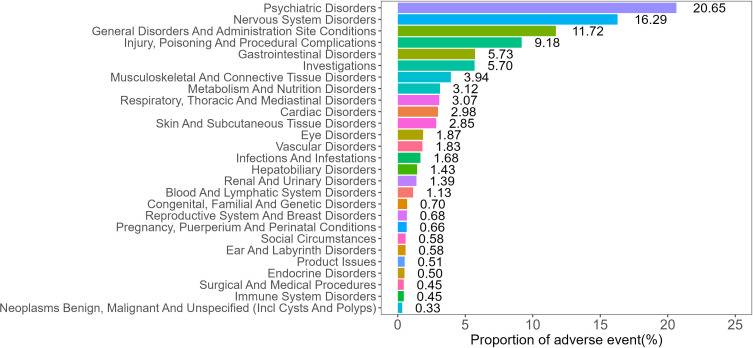
Distribution of AEs at the SOC level for mirtazapine.

AEs related to mirtazapine were distributed across 27 SOCs, and their proportions are shown in Fig 2. Two SOCs demonstrated positive signals, namely psychiatric disorders (n = 12,481, ROR = 4.35, PRR = 3.66, EBGM = 3.65, IC = 1.87) and congenital, familial and genetic disorders (n = 421, ROR = 2.32, PRR = 2.31, EBGM = 2.31, IC = 1.21). The signal values for all SOCs are shown in Table 2.

**Table 2:** Signal strength of mirtazapine AEs across SOC in the FAERS database..

SOC	Numbers	ROR(95%CI)	PRR(χ^2^)	EBGM(EBGM05)	IC(IC025)
Psychiatric disorders*	12,481	4.35 (4.27 - 4.44)	3.66 (25451.63)	3.65 (3.58)	1.87 (1.84)
Nervous system disorders	9,844	2.11 (2.07 - 2.16)	1.93 (4802.63)	1.93 (1.89)	0.95 (0.91)
General disorders and administration site conditions	7,084	0.63 (0.61 - 0.64)	0.67 (1368.19)	0.67 (0.66)	−0.57 (−0.61)
Injury, poisoning and procedural complications	5,548	0.86 (0.84 - 0.89)	0.87 (111.54)	0.87 (0.85)	−0.19 (−0.23)
Gastrointestinal disorders	3,460	0.65 (0.63 - 0.68)	0.67 (594.82)	0.67 (0.65)	−0.57 (−0.62)
Investigations	3,443	0.93 (0.9 - 0.96)	0.93 (16.61)	0.93 (0.9)	−0.1 (−0.15)
Musculoskeletal and connective tissue disorders	2,383	0.75 (0.72 - 0.78)	0.76 (188.44)	0.76 (0.73)	−0.39 (−0.45)
Metabolism and nutrition disorders	1,883	1.45 (1.38 - 1.52)	1.44 (253.6)	1.43 (1.37)	0.52 (0.45)
Respiratory, thoracic and mediastinal disorders	1,854	0.64 (0.61 - 0.67)	0.65 (357.14)	0.65 (0.62)	−0.61 (−0.68)
Cardiac disorders	1,801	1.14 (1.09 - 1.19)	1.14 (29.77)	1.14 (1.08)	0.18 (0.11)
Skin and subcutaneous tissue disorders	1,723	0.51 (0.49 - 0.53)	0.52 (790.09)	0.52 (0.5)	−0.93 (−1)
Eye disorders	1,129	0.94 (0.88 - 1)	0.94 (4.49)	0.94 (0.89)	−0.09 (−0.18)
Vascular disorders	1,107	0.86 (0.81 - 0.91)	0.86 (24.44)	0.86 (0.81)	−0.21 (−0.3)
Infections and infestations	1,016	0.31 (0.29 - 0.33)	0.32 (1522.55)	0.32 (0.3)	−1.63 (−1.72)
Hepatobiliary disorders	865	1.57 (1.46 - 1.68)	1.56 (174.28)	1.56 (1.46)	0.64 (0.54)
Renal and urinary disorders	837	0.73 (0.68 - 0.78)	0.74 (80.6)	0.74 (0.69)	−0.44 (−0.54)
Blood and lymphatic system disorders	683	0.67 (0.62 - 0.72)	0.67 (111.74)	0.67 (0.62)	−0.57 (−0.68)
Congenital, familial and genetic disorders*	421	2.32 (2.11 - 2.55)	2.31 (312.93)	2.31 (2.1)	1.21 (1.06)
Reproductive system and breast disorders	411	0.77 (0.7 - 0.85)	0.77 (28.63)	0.77 (0.7)	−0.38 (−0.52)
Pregnancy, puerperium and perinatal conditions	399	1.57 (1.42 - 1.73)	1.56 (81.24)	1.56 (1.42)	0.64 (0.5)
Social circumstances	352	1.24 (1.12 - 1.38)	1.24 (16.14)	1.24 (1.11)	0.31 (0.15)
Ear and labyrinth disorders	352	1.35 (1.21 - 1.5)	1.35 (31.39)	1.35 (1.21)	0.43 (0.27)
Product issues	307	0.31 (0.28 - 0.34)	0.31 (474.98)	0.31 (0.28)	−1.68 (−1.84)
Endocrine disorders	305	1.97 (1.76 - 2.21)	1.97 (145.72)	1.97 (1.76)	0.98 (0.81)
Surgical and medical procedures	271	0.33 (0.29 - 0.37)	0.33 (376.55)	0.33 (0.29)	−1.6 (−1.78)
Immune system disorders	269	0.4 (0.36 - 0.45)	0.4 (239.6)	0.4 (0.36)	−1.31 (−1.48)
Neoplasms benign, malignant and unspecified (incl cysts and polyps)	201	0.12 (0.11 - 0.14)	0.13 (1227.53)	0.13 (0.11)	−2.97 (−3.16)

Abbreviation: Asterisks (*) indicate statistically significant signals in algorithm; ROR, reporting odds ratio; PRR, proportional reporting ratio; EBGM, empirical Bayesian geometric mean; EBGM05, the lower limit of the 95% CI of EBGM; IC, information component; IC025, the lower limit of the 95% CI of the IC; CI, confidence interval; AEs, adverse events.

### Distribution of AEs across PT

**Fig 3:**
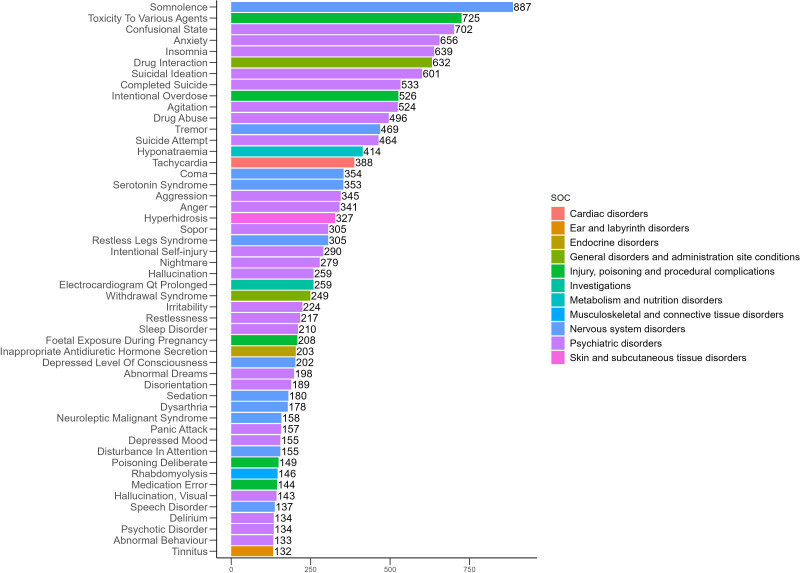
Distribution of the top 50 positive AEs by frequency at the PT level for mirtazapine.

**Fig 4:**
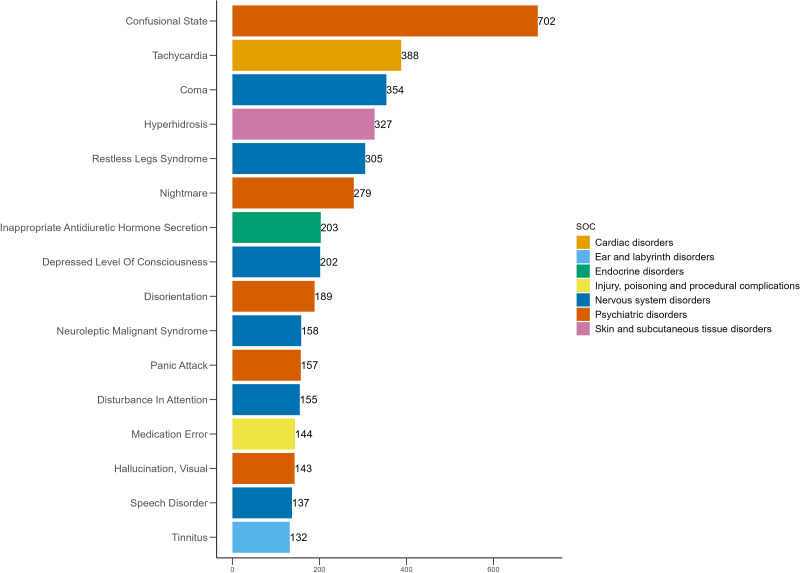
Unexpected AEs with positive signals among the top 50 by frequency at the PT level for mirtazapine.

The 50 most frequently reported positive AEs and their signal strengths are summarized in Table 3, and their distribution is illustrated in Fig 3. The five most common AEs with positive signals were somnolence (n = 887, ROR = 4.57, PRR = 4.52, EBGM = 4.50, IC = 2.17), toxicity to various agents (n = 725, ROR = 4.07, PRR = 4.04, EBGM = 4.02, IC = 2.01), confusional state (n = 702, ROR = 4.45, PRR = 4.41, EBGM = 4.39, IC = 2.13), anxiety (n = 656, ROR = 2.31, PRR = 2.30, EBGM = 2.29, IC = 1.20) and insomnia (n = 639, ROR = 2.43, PRR = 2.41, EBGM = 2.41, IC = 1.27). This study confirmed several known AEs of mirtazapine with positive signals, including somnolence (n = 887, ROR = 4.57, PRR = 4.52, EBGM = 4.50, IC = 2.17), suicidal ideation (n = 601, ROR = 6.65, PRR = 6.59, EBGM = 6.55, IC = 2.71), tremor (n = 469, ROR = 2.86, PRR = 2.85, EBGM = 2.84, IC = 1.51), hyponatraemia (n = 414, ROR = 7.62, PRR = 7.57, EBGM = 7.51, IC = 2.91), QT interval prolongation on electrocardiogram (n = 259, ROR = 7.35, PRR = 7.32, EBGM = 7.27, IC = 2.86) and rhabdomyolysis (n = 146, ROR = 3.66, PRR = 3.65, EBGM = 3.64, IC = 1.87). In addition, several unexpected positive AEs were identified, including confusional state (n = 702, ROR = 4.45, PRR = 4.41, EBGM = 4.39, IC = 2.13), tachycardia (n = 388, ROR = 4.52, PRR = 4.50, EBGM = 4.48, IC = 2.16), coma (n = 354, ROR = 7.85, PRR = 7.81, EBGM = 7.75, IC = 2.95), restless legs syndrome (n = 305, ROR = 17.05, PRR = 16.97, EBGM = 16.65, IC = 4.06), nightmare (n = 279, ROR = 8.02, PRR = 7.99, EBGM = 7.93, IC = 2.99), inappropriate antidiuretic hormone secretion (n = 203, ROR = 21.91, PRR = 21.84, EBGM = 21.31, IC = 4.41), disorientation (n = 189, ROR = 4.78, PRR = 4.77, EBGM = 4.75, IC = 2.25), neuroleptic malignant syndrome (n = 158, ROR = 13.77, PRR = 13.74, EBGM = 13.53, IC = 3.76) and tinnitus (n = 132, ROR = 2.94, PRR = 2.94, EBGM = 2.93, IC = 1.55). The distribution of these unexpected positive AEs is shown in Fig 4.

**Table 3:** Top 50 positive AEs associated with mirtazapine at the PT level.

PT	Numbers	ROR(95%CI)	PRR(χ^2^)	EBGM(EBGM05)	IC(IC025)
Somnolence	887	4.57 (4.28 - 4.89)	4.52 (2427.99)	4.5 (4.21)	2.17 (2.07)
Toxicity to various agents	725	4.07 (3.78 - 4.38)	4.04 (1652.49)	4.02 (3.74)	2.01 (1.89)
Confusional state	702	4.45 (4.13 - 4.79)	4.41 (1845.58)	4.39 (4.08)	2.13 (2.02)
Anxiety	656	2.31 (2.14 - 2.5)	2.3 (481.66)	2.29 (2.12)	1.2 (1.08)
Insomnia	639	2.43 (2.25 - 2.62)	2.41 (529.31)	2.41 (2.23)	1.27 (1.15)
Drug interaction	632	4.09 (3.79 - 4.43)	4.06 (1455.5)	4.05 (3.74)	2.02 (1.89)
Suicidal ideation	601	6.65 (6.13 - 7.2)	6.59 (2831.79)	6.55 (6.04)	2.71 (2.58)
Completed suicide	533	6.59 (6.05 - 7.18)	6.54 (2486.32)	6.5 (5.97)	2.7 (2.56)
Intentional overdose	526	8.28 (7.6 - 9.03)	8.22 (3306.08)	8.15 (7.48)	3.03 (2.88)
Agitation	524	7.19 (6.6 - 7.84)	7.14 (2745.73)	7.09 (6.5)	2.83 (2.68)
Drug abuse	496	6.19 (5.66 - 6.76)	6.14 (2123.65)	6.11 (5.59)	2.61 (2.47)
Tremor	469	2.86 (2.61 - 3.13)	2.85 (561.1)	2.84 (2.59)	1.51 (1.37)
Suicide attempt	464	7.85 (7.16 - 8.6)	7.8 (2725.71)	7.73 (7.05)	2.95 (2.8)
Hyponatraemia	414	7.62 (6.91 - 8.39)	7.57 (2342.29)	7.51 (6.82)	2.91 (2.74)
Tachycardia	388	4.52 (4.09 - 5)	4.5 (1051.98)	4.48 (4.05)	2.16 (2)
Coma	354	7.85 (7.07 - 8.72)	7.81 (2084.94)	7.75 (6.98)	2.95 (2.77)
Serotonin syndrome	353	19.68 (17.7 - 21.88)	19.57 (6079.83)	19.15 (17.22)	4.26 (4.03)
Aggression	345	6.92 (6.22 - 7.69)	6.88 (1722.48)	6.84 (6.15)	2.77 (2.59)
Anger	341	9.99 (8.98 - 11.12)	9.94 (2711.65)	9.84 (8.84)	3.3 (3.1)
Hyperhidrosis	327	2.57 (2.31 - 2.87)	2.56 (311.08)	2.56 (2.29)	1.35 (1.19)
Sopor	305	23.36 (20.84 - 26.18)	23.25 (6318.99)	22.64 (20.2)	4.5 (4.23)
Restless legs syndrome	305	17.05 (15.22 - 19.1)	16.97 (4492.65)	16.65 (14.86)	4.06 (3.82)
Intentional self-injury	290	14.05 (12.51 - 15.78)	13.99 (3440.72)	13.77 (12.26)	3.78 (3.55)
Nightmare	279	8.02 (7.13 - 9.03)	7.99 (1691.59)	7.93 (7.04)	2.99 (2.78)
Hallucination	259	3.61 (3.19 - 4.07)	3.59 (483.41)	3.58 (3.17)	1.84 (1.65)
Electrocardiogram qt prolonged	259	7.35 (6.5 - 8.31)	7.32 (1402.67)	7.27 (6.43)	2.86 (2.65)
Withdrawal syndrome	249	5.81 (5.13 - 6.58)	5.79 (980.9)	5.76 (5.08)	2.53 (2.31)
Irritability	224	3.69 (3.23 - 4.21)	3.68 (435.59)	3.67 (3.22)	1.87 (1.66)
Restlessness	217	6 (5.25 - 6.86)	5.98 (894.09)	5.94 (5.2)	2.57 (2.34)
Sleep disorder	210	3.17 (2.77 - 3.63)	3.16 (309.13)	3.15 (2.75)	1.66 (1.44)
Foetal exposure during pregnancy	208	2.58 (2.25 - 2.96)	2.57 (199.97)	2.57 (2.24)	1.36 (1.15)
Inappropriate antidiuretic hormone secretion	203	21.91 (19.06 - 25.2)	21.84 (3935.33)	21.31 (18.54)	4.41 (4.07)
Depressed level of consciousness	202	5.4 (4.7 - 6.2)	5.38 (716.32)	5.35 (4.66)	2.42 (2.19)
Abnormal dreams	198	6.9 (6 - 7.94)	6.88 (987.74)	6.83 (5.94)	2.77 (2.53)
Disorientation	189	4.78 (4.15 - 5.52)	4.77 (560.68)	4.75 (4.12)	2.25 (2.01)
Sedation	180	7.56 (6.53 - 8.76)	7.54 (1012.61)	7.48 (6.46)	2.9 (2.64)
Dysarthria	178	4.79 (4.14 - 5.55)	4.78 (529.62)	4.76 (4.11)	2.25 (2)
Neuroleptic malignant syndrome	158	13.77 (11.77 - 16.12)	13.74 (1836.74)	13.53 (11.56)	3.76 (3.42)
Panic attack	157	4.36 (3.73 - 5.1)	4.35 (403.14)	4.33 (3.7)	2.12 (1.85)
Depressed mood	155	3.03 (2.59 - 3.55)	3.03 (209.78)	3.02 (2.58)	1.59 (1.34)
Disturbance in attention	155	2.85 (2.43 - 3.33)	2.84 (184.64)	2.84 (2.42)	1.5 (1.26)
Poisoning deliberate	149	18.88 (16.05 - 22.21)	18.83 (2461)	18.44 (15.67)	4.2 (3.81)
Rhabdomyolysis	146	3.66 (3.11 - 4.31)	3.65 (280.49)	3.64 (3.1)	1.87 (1.6)
Medication error	144	2.81 (2.38 - 3.3)	2.8 (166.37)	2.8 (2.37)	1.48 (1.22)
Hallucination, visual	143	7.25 (6.15 - 8.55)	7.24 (762.49)	7.18 (6.09)	2.84 (2.54)
Speech disorder	137	2.65 (2.24 - 3.13)	2.64 (139.57)	2.64 (2.23)	1.4 (1.14)
Delirium	134	4.02 (3.39 - 4.76)	4.01 (301.7)	4 (3.37)	2 (1.72)
Psychotic disorder	134	4.6 (3.89 - 5.46)	4.6 (375.24)	4.58 (3.86)	2.19 (1.91)
Abnormal behaviour	133	3.28 (2.77 - 3.89)	3.28 (209.96)	3.27 (2.76)	1.71 (1.43)
Tinnitus	132	2.94 (2.48 - 3.49)	2.94 (168.35)	2.93 (2.47)	1.55 (1.28)

Abbreviation: ROR, reporting odds ratio; PRR, proportional reporting ratio; EBGM, empirical Bayesian geometric mean; EBGM05, the lower limit of the 95% CI of EBGM; IC, information component; IC025, the lower limit of the 95% CI of the IC; CI, confidence interval; PT, preferred term.

### Sensitivity analysis

**Fig 5:**
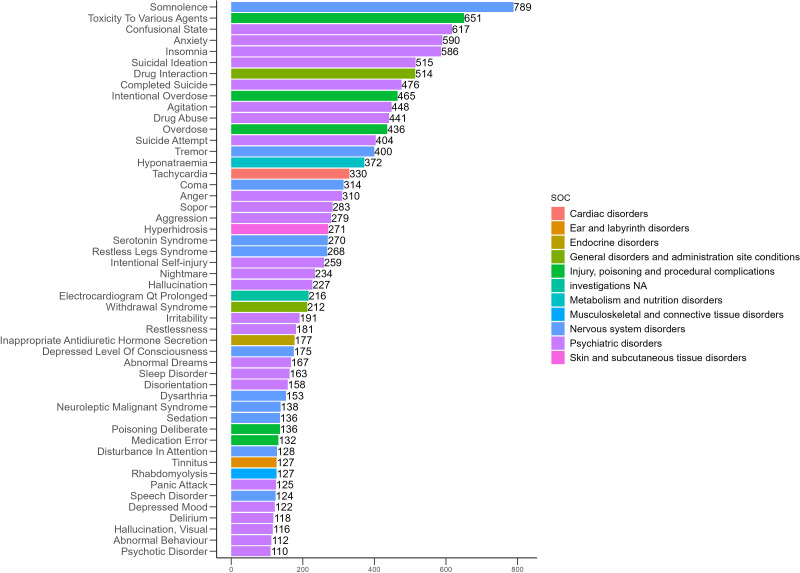
Distribution of the top 50 positive AEs by frequency at the PT level for mirtazapine after excluding common concomitant medications.

**Fig 6:**
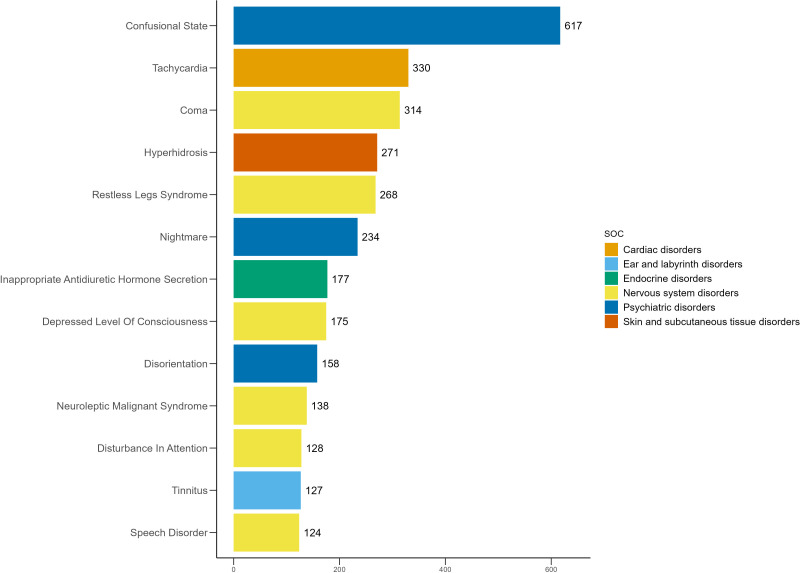
Unexpected AEs with positive signals among the top 50 by frequency at the PT level for mirtazapine after excluding common concomitant medications.

After excluding reports involving concomitant use with mirtazapine, a new disproportionality analysis was conducted. Table 4 presents the signal strength of the top 50 most frequently reported positive AEs. The distribution of these AEs is shown in Fig 5, and the unexpected positive AEs are illustrated in Fig 6. The five most common AEs with positive signals were somnolence (n = 789, ROR = 4.69, PRR = 4.64, EBGM = 4.62, IC = 2.10), toxicity to various agents (n = 651, ROR = 4.22, PRR = 4.18, EBGM = 4.16, IC = 2.06), confusional state (n = 617, ROR = 4.51, PRR = 4.47, EBGM = 4.45, IC = 2.15), anxiety (n = 590, ROR = 2.40, PRR = 2.38, EBGM = 2.38, IC = 1.25) and insomnia (n = 586, ROR = 2.57, PRR = 2.55, EBGM = 2.55, IC = 1.35). In addition, somnolence (n = 789, ROR = 4.69, PRR = 4.64, EBGM = 4.62, IC = 2.10), suicidal ideation (n = 515, ROR = 6.56, PRR = 6.51, EBGM = 6.47, IC = 2.69), tremor (n = 400, ROR = 2.81, PRR = 2.80, EBGM = 2.79, IC = 1.48), hyponatraemia (n = 372, ROR = 7.89, PRR = 7.84, EBGM = 7.79, IC = 2.96), QT interval prolongation on electrocardiogram (n = 216, ROR = 7.06, PRR = 7.04, EBGM = 6.99, IC = 2.81), rhabdomyolysis (n = 127, ROR = 3.67, PRR = 3.67, EBGM = 3.66, IC = 1.87), confusional state (n = 617, ROR = 4.51, PRR = 4.47, EBGM = 4.45, IC = 2.15), tachycardia (n = 330, ROR = 4.43, PRR = 4.41, EBGM = 4.40, IC = 2.14), coma (n = 314, ROR = 8.03, PRR = 7.99, EBGM = 7.93, IC = 2.99), restless legs syndrome (n = 305, ROR = 17.05, PRR = 16.97, EBGM = 16.65, IC = 4.06), nightmare (n = 279, ROR = 8.02, PRR = 7.99, EBGM = 7.93, IC = 2.99), inappropriate antidiuretic hormone secretion (n = 203, ROR = 21.91, PRR = 21.84, EBGM = 21.31, IC = 4.41), disorientation (n = 189, ROR = 4.78, PRR = 4.77, EBGM = 4.75, IC = 2.25), neuroleptic malignant syndrome (n = 158, ROR = 13.85, PRR = 13.82, EBGM = 13.64, IC = 3.77) and tinnitus (n = 127, ROR = 3.27, PRR = 3.26, EBGM = 3.25, IC = 1.70) continued to show positive signals. The results from the sensitivity analysis further supported the findings from the primary analysis.

**Table 4:** Top 50 positive AEs for mirtazapine excluding common medication co-usage at the PT level.

PT	Numbers	ROR(95%CI)	PRR(χ^2^)	EBGM(EBGM05)	IC(IC025)
Somnolence	789	4.69 (4.37 - 5.04)	4.64 (2247.8)	4.62 (4.31)	2.21 (2.1)
Toxicity to various agents	651	4.22 (3.9 - 4.56)	4.18 (1571.9)	4.16 (3.85)	2.06 (1.94)
Confusional state	617	4.51 (4.16 - 4.88)	4.47 (1657.47)	4.45 (4.11)	2.15 (2.03)
Anxiety	590	2.4 (2.21 - 2.6)	2.38 (474.74)	2.38 (2.19)	1.25 (1.13)
Insomnia	586	2.57 (2.37 - 2.79)	2.55 (553.98)	2.55 (2.35)	1.35 (1.23)
Suicidal ideation	515	6.56 (6.01 - 7.16)	6.51 (2387.95)	6.47 (5.93)	2.69 (2.55)
Drug interaction	514	3.84 (3.52 - 4.18)	3.81 (1062.74)	3.8 (3.48)	1.92 (1.79)
Completed suicide	476	6.79 (6.2 - 7.43)	6.73 (2311.31)	6.69 (6.12)	2.74 (2.59)
Intentional overdose	465	8.44 (7.7 - 9.25)	8.37 (2995.91)	8.31 (7.58)	3.05 (2.9)
Agitation	448	7.08 (6.45 - 7.78)	7.03 (2304)	6.99 (6.37)	2.8 (2.65)
Drug abuse	441	6.34 (5.77 - 6.97)	6.3 (1955.07)	6.26 (5.7)	2.65 (2.49)
Overdose	436	2.22 (2.02 - 2.44)	2.21 (289.22)	2.21 (2.01)	1.14 (1)
Suicide attempt	404	7.87 (7.14 - 8.69)	7.82 (2386.15)	7.77 (7.04)	2.96 (2.79)
Tremor	400	2.81 (2.55 - 3.1)	2.8 (462.3)	2.79 (2.53)	1.48 (1.33)
Hyponatraemia	372	7.89 (7.12 - 8.74)	7.84 (2204.65)	7.79 (7.03)	2.96 (2.78)
Tachycardia	330	4.43 (3.98 - 4.94)	4.41 (867.9)	4.4 (3.94)	2.14 (1.96)
Coma	314	8.03 (7.18 - 8.97)	7.99 (1904.53)	7.93 (7.09)	2.99 (2.79)
Anger	310	10.47 (9.36 - 11.72)	10.42 (2611.73)	10.31 (9.22)	3.37 (3.16)
Sopor	283	24.96 (22.18 - 28.1)	24.84 (6312.3)	24.24 (21.53)	4.6 (4.31)
Aggression	279	6.44 (5.72 - 7.25)	6.41 (1267.28)	6.38 (5.67)	2.67 (2.47)
Hyperhidrosis	271	2.46 (2.18 - 2.77)	2.45 (232.03)	2.44 (2.17)	1.29 (1.11)
Serotonin syndrome	270	17.26 (15.3 - 19.47)	17.17 (4041.98)	16.89 (14.97)	4.08 (3.82)
Restless legs syndrome	268	17.24 (15.27 - 19.46)	17.16 (4007.28)	16.87 (14.95)	4.08 (3.82)
Intentional self-injury	259	14.45 (12.78 - 16.34)	14.39 (3179.64)	14.19 (12.55)	3.83 (3.57)
Nightmare	234	7.75 (6.81 - 8.82)	7.72 (1359.01)	7.67 (6.74)	2.94 (2.71)
Hallucination	227	3.64 (3.2 - 4.15)	3.63 (431.86)	3.62 (3.18)	1.86 (1.65)
Electrocardiogram qt prolonged	216	7.06 (6.17 - 8.07)	7.04 (1110.97)	6.99 (6.11)	2.81 (2.57)
Withdrawal syndrome	212	5.7 (4.98 - 6.53)	5.68 (813.75)	5.65 (4.94)	2.5 (2.27)
Irritability	191	3.63 (3.15 - 4.18)	3.62 (360.68)	3.61 (3.13)	1.85 (1.62)
Restlessness	181	5.76 (4.98 - 6.67)	5.75 (706.01)	5.72 (4.94)	2.52 (2.26)
Inappropriate antidiuretic hormone secretion	177	21.97 (18.92 - 25.51)	21.9 (3452.35)	21.43 (18.46)	4.42 (4.05)
Depressed level of consciousness	175	5.39 (4.64 - 6.25)	5.37 (619.84)	5.35 (4.61)	2.42 (2.17)
Abnormal dreams	167	6.71 (5.76 - 7.81)	6.69 (802.64)	6.65 (5.71)	2.73 (2.46)
Sleep disorder	163	2.83 (2.43 - 3.3)	2.83 (192.08)	2.82 (2.42)	1.5 (1.25)
Disorientation	158	4.61 (3.94 - 5.39)	4.6 (443.01)	4.58 (3.92)	2.2 (1.93)
Dysarthria	153	4.75 (4.05 - 5.57)	4.74 (449.22)	4.72 (4.03)	2.24 (1.97)
Neuroleptic malignant syndrome	138	13.85 (11.71 - 16.39)	13.82 (1617.86)	13.64 (11.52)	3.77 (3.4)
Sedation	136	6.57 (5.55 - 7.78)	6.56 (636.69)	6.52 (5.51)	2.71 (2.4)
Poisoning deliberate	136	19.84 (16.74 - 23.52)	19.79 (2378.2)	19.41 (16.38)	4.28 (3.85)
Medication error	132	2.97 (2.5 - 3.52)	2.96 (171.11)	2.96 (2.49)	1.56 (1.29)
Disturbance in attention	128	2.71 (2.28 - 3.22)	2.71 (137.44)	2.7 (2.27)	1.43 (1.16)
Tinnitus	127	3.27 (2.74 - 3.89)	3.26 (198.6)	3.25 (2.73)	1.7 (1.42)
Rhabdomyolysis	127	3.67 (3.08 - 4.37)	3.67 (245.39)	3.66 (3.07)	1.87 (1.58)
Panic attack	125	4 (3.35 - 4.77)	3.99 (279.21)	3.98 (3.34)	1.99 (1.7)
Speech disorder	124	2.76 (2.32 - 3.3)	2.76 (138.74)	2.75 (2.31)	1.46 (1.18)
Depressed mood	122	2.75 (2.3 - 3.29)	2.75 (135.2)	2.74 (2.29)	1.45 (1.17)
Delirium	118	4.08 (3.4 - 4.89)	4.07 (272.61)	4.06 (3.39)	2.02 (1.72)
Hallucination, visual	116	6.78 (5.64 - 8.13)	6.76 (565.86)	6.72 (5.6)	2.75 (2.41)
Abnormal behaviour	112	3.19 (2.65 - 3.84)	3.18 (167.28)	3.18 (2.64)	1.67 (1.37)
Psychotic disorder	110	4.36 (3.61 - 5.25)	4.35 (282.55)	4.33 (3.59)	2.12 (1.8)

Abbreviation: ROR, reporting odds ratio; PRR, proportional reporting ratio; EBGM, empirical Bayesian geometric mean; EBGM05, the lower limit of the 95% CI of EBGM; IC, information component; IC025, the lower limit of the 95% CI of the IC; CI, confidence interval; PT, preferred term.

### Age subgroup analysis

Among individuals younger than 18 years, suicide attempt was the most common positive suicide-related AE (S3 Table). In the 18–64 years group, suicidal ideation, completed suicide and suicide attempt were the most prominent and frequently reported positive suicide-related AEs (S4 Table). In patients aged ≥65 years, intentional self-injury and suicide attempt were the most common (S5 Table).

### TTO and Weibull distribution

**Fig 7:**
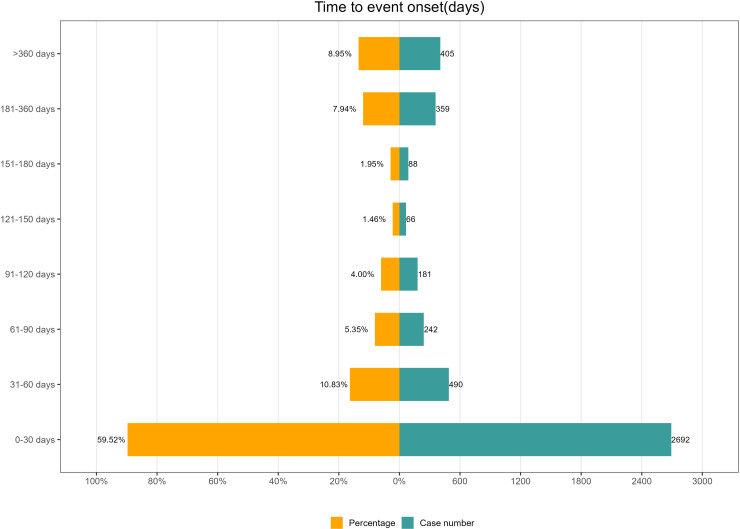
Time to onset distribution of mirtazapine-related AEs.

**Fig 8:**
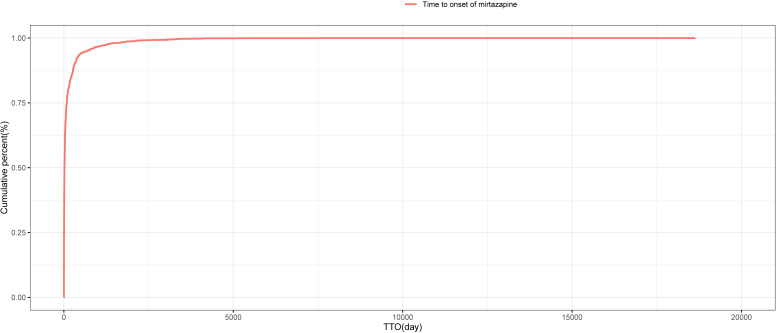
Cumulative incidence of mirtazapine-related AEs over time.

A total of 4,523 reports contained valid TTO data for mirtazapine-related AEs. The median TTO was 18 days. The Weibull distribution indicated an early failure model, with detailed shape and scale parameters provided in Table 5. Fig 7 shows the distribution of TTOs, with 59.52% of AEs occurring within the first month of drug exposure. The cumulative incidence of AEs over time is illustrated in Fig 8.

**Table 5:** Time to onset of mirtazapine-associated adverse events and Weibull distribution analysis.

Drug	TTO(days)		Weibull distribution		
	Case reports	Median(d)(IQR)	Scale parameter: α(95%CI)	Shape parameter: β(95%CI)	Type
Mirtazapine	4523	18(4.00,86.50)	57.10(53.50,60.70)	0.49(0.48,0.50)	Early failure

Abbreviation: TTO,time to onset; CI, confidence interval; IQR, interquartile range.

## Disscussion

Mirtazapine remains widely used in the clinical treatment of MDD, yet real-world safety evaluations are still limited. We analyzed mirtazapine-associated AE reports from the FAERS database spanning from Q1 2004 to Q1 2025 using four disproportionality analysis methods. Several known AEs were confirmed to exhibit positive signals, including somnolence, suicidal ideation, hyponatraemia, QT interval prolongation on electrocardiogram, and rhabdomyolysis. In addition, several unexpected AEs also showed positive signals, such as confusional state, tachycardia, coma, restless legs syndrome, nightmare, inappropriate antidiuretic hormone secretion, disorientation, neuroleptic malignant syndrome, and tinnitus.

Somnolence is a common AE. In a randomized controlled trial, Claghorn et al. reported a significantly higher incidence of somnolence in the mirtazapine group than in the placebo group. The incidence reached 62% in the mirtazapine group [18]. Another review also confirmed that somnolence is one of the most frequently reported AEs of mirtazapine [6]. In our study, a significant association between mirtazapine use and somnolence was identified. Clinically, somnolence presents as excessive daytime sleepiness, decreased alertness, and cognitive sluggishness, which may severely impact patients’ daily functioning and quality of life [19,20]. This AE is primarily attributed to the strong antagonism of central histamine H₁ receptors by mirtazapine [5]. Recommended strategies include bedtime administration, gradual dose titration, and comprehensive patient education regarding potential sedative effects. For patients who cannot tolerate this AE, switching to a less sedating antidepressant may be warranted.

In addition, this study revealed a potential association between mirtazapine and QT interval prolongation. Although the overall incidence with mirtazapine is relatively low, its potential severity should not be overlooked. Several case reports have suggested that mirtazapine may trigger QT prolongation [21]. Therefore, increased vigilance is warranted in high-risk patients, especially those with underlying cardiovascular conditions. For patients at high risk of QT prolongation, cautious use or consideration of alternative antidepressants may be necessary.

This study identified a potential signal linking mirtazapine with neuroleptic malignant syndrome (NMS). NMS is a rare but potentially fatal AE traditionally associated with dopamine receptor antagonists, particularly typical antipsychotics [22]. As an antidepressant with a unique pharmacological profile, mirtazapine has not previously been listed in drug labeling as being associated with NMS. Clinically, NMS is characterized by a classic tetrad of hyperthermia, generalized muscle rigidity, autonomic dysfunction, and altered mental status, which can be easily misdiagnosed as severe infection, serotonin syndrome, or catatonia [23,24]. Moreover, several published case reports have documented NMS associated with mirtazapine [25,26]. Given the severity of NMS, clinicians should remain vigilant when prescribing mirtazapine in polypharmacy contexts or in patients with known risk factors such as dehydration or underlying neurological disorders.

Finally, we identified several unexpected neuropsychiatric AEs potentially associated with mirtazapine use, including nightmare, depressed level of consciousness, disorientation, and disturbance in attention. Although these AEs are not prominently listed in the product labeling, they may reflect under-recognized manifestations of mirtazapine’s effects on the central nervous system. The clinical severity of these symptoms varies, but in vulnerable populations such as the elderly, they may interfere with daily functioning and reduce medication adherence.

Mirtazapine is often co-administered with other medications in clinical practice, which may potentially confound the findings of this study. To address this, we excluded reports involving the concomitant use of mirtazapine with commonly prescribed drugs and re-conducted the disproportionality analysis. The results showed that AEs such as somnolence, rhabdomyolysis, QT prolongation, restless legs syndrome, and neuroleptic malignant syndrome continued to demonstrate positive signals, supporting the robustness of our findings.

A pharmacovigilance analysis of prescription-related AEs in 13,554 patients in the UK identified drowsiness and lassitude as the most commonly occurring AEs, with abnormal dreams also noted [27]. These findings are similar to the somnolence and nightmare events identified in our study. From a pharmacological perspective, the receptor profile of mirtazapine, characterised by antagonism at central α2 receptors, enhancement of 5HT and NE neurotransmission and blockade of H1 receptors, can largely explain some of the positive signals observed in this study, such as somnolence. Additionally, a pharmacovigilance study on antidepressants and arrhythmias highlighted an association between mirtazapine and heart block [28]. Our study also observed cardiac AEs associated with mirtazapine, such as QT interval prolongation and tachycardia. These findings suggest potential abnormalities related to both the neuropsychiatric and cardiac effects of mirtazapine, indicating that these AEs should be closely monitored in future clinical use to ensure patient safety.

Although the patterns of suicide-related AEs differed across age groups, patients in all age categories warrant careful attention. In individuals younger than 18 years, suicide attempt was the most common suicide-related AE. Among adults aged 18–64 years, the higher numbers of suicidal ideation, completed suicide and suicide attempt reflect the psychological risks that may accompany pharmacological treatment, especially in patients with depressive disorders. In older adults (≥65 years), intentional self-injury and suicide attempt also emerged as important positive signals. Taken together, despite age-specific differences in the presentation of suicide-related AEs, enhanced monitoring of suicide risk should be implemented for patients of all ages [29], particularly during the initial phase of treatment or when adjusting the dose. Timely identification and appropriate management of suicide-related AEs are essential to improving patient safety.

The TTO analysis showed that 59.5% of AEs occurred within the first month after mirtazapine administration, with a median onset time of 18 days. The Weibull distribution model indicated a decreasing hazard over time, suggesting that AEs tend to occur earlier in the course of treatment. This further supports our current findings. These results highlight the need for enhanced safety monitoring during the initial phase of mirtazapine therapy.

Pharmacovigilance constitutes a core component of the evaluation and continuous management of drug safety across the life cycle of a medicinal product. Given the widespread and long term use of drugs in real world settings, rare, unexpected, or delayed AEs not fully captured in clinical trials are more likely to be identified through pharmacovigilance systems. Systematic signal detection and risk assessment provide essential support for timely recognition of safety concerns and for guiding regulatory updates and clinical decision making [30]. For mirtazapine, ongoing pharmacovigilance activities contribute to a more complete understanding of its neuropsychiatric and cardiovascular safety profile and assist in balancing therapeutic benefits and risks in practice. Continued efforts to enhance pharmacovigilance research using real world data and structured signal analysis will help refine the long term safety evidence for psychotropic medications and other widely used drugs.

This study has several limitations. First, as a spontaneous reporting system, the FAERS database is subject to inherent biases such as underreporting, duplicate submissions, and variability in report quality, which may affect the accuracy and completeness of the data [31]. Second, due to the lack of denominator data, detailed clinical background, and information on concomitant medications, it is not possible to establish definitive causal relationships between mirtazapine and the observed AEs. Third, the signal detection methods applied are designed to identify potential associations but are not suitable for inferring causal relationships [32]. Finally, although we excluded some reports involving concomitant drug use in the sensitivity analysis, residual confounding cannot be completely ruled out. Therefore, the findings should be interpreted with caution and warrant further validation through prospective studies or mechanistic research.

This study has several strengths. First, it analyzed more than 20 years of mirtazapine-related AE reports, providing a comprehensive long-term safety perspective. Second, the use of real-world pharmacovigilance data complements the limitations of clinical trials, particularly in capturing rare, unexpected or delayed AEs. Third, four disproportionality analysis methods were applied, and only AEs that met the criteria of all four algorithms were defined as positive signals, thereby improving the robustness of the results. Finally, to further enhance reliability, we included only reports in which mirtazapine was identified as the primary suspect drug and conducted a sensitivity analysis to verify the stability of the findings.

## Conclusion

In summary, this study systematically evaluated the real-world safety profile of mirtazapine based on 21 years of data from the FAERS database. The results not only confirmed known AEs such as somnolence and QT interval prolongation, but also revealed potential associations with rare but serious events including rhabdomyolysis and neuroleptic malignant syndrome. Additionally, several unexpected neuropsychiatric AEs were identified, such as nightmare, disorientation, and disturbance in attention. This study underscores the importance of ongoing pharmacovigilance for mirtazapine and provides valuable insights to support safer and more individualized antidepressant therapy. Further research is needed to validate these findings and explore the underlying mechanisms.

## Supporting information

S1 Table Two-by-two contingency table for disproportionality analyses. (DOCX)

S2 Table Four major algorithms used for signal detection. (DOCX)

S3 Table Top 50 positive AEs associated with mirtazapine at the PT level in patients younger than 18 years. (DOCX)

S4 Table Top 50 positive AEs associated with mirtazapine at the PT level in patients aged 18–64 years. (DOCX)

S5 Table Top 50 positive AEs associated with mirtazapine at the PT level in patients aged ≥65 years. (DOCX)
