# Supplementary material for: Long-term safety evaluation of mirtazapine: A real-world pharmacovigilance study based on the FAERS database
Source: PLoS One. 2026 Mar 6;21(3):e0340092. doi: 10.1371/journal.pone.0340092 (PMC12965596; doi:10.1371/journal.pone.0340092)
Supplement: S1 Table — (DOCX) [file pone.0340092.s001.docx]

Supplementary Table1

**Two-by-two contingency table for disproportionality analyses**

|  | Target AEs | Other AEs | Total |
| --- | --- | --- | --- |
| Mirtazapine | a | b | a+b |
| Other drugs | c | d | c+d |
| Total | a+c | b+d | a+b+c+d |

Abbreviation: AEs, adverse events; a, number of reports containing both the target drug and target adverse drug reaction; b, number of reports containing other adverse drug reaction of the target drug; c, number of reports containing the target adverse drug reaction of other drugs; d, number of reports containing other drugs and other adverse drug reactions.
