# Supplementary material for: Long-term safety evaluation of mirtazapine: A real-world pharmacovigilance study based on the FAERS database
Source: PLoS One. 2026 Mar 6;21(3):e0340092. doi: 10.1371/journal.pone.0340092 (PMC12965596; doi:10.1371/journal.pone.0340092)
Supplement: S4 Table — (DOCX) [file pone.0340092.s004.docx]

**Supplementary Table4**

**Top 50 positive AEs associated with mirtazapine at the PT level in patients aged 18–64 years**

| PT | Numbers | ROR(95%CI) | PRR(χ^2^) | EBGM(EBGM05) | IC(IC025) |
| --- | --- | --- | --- | --- | --- |
| Toxicity to various agents | 525 | 4.48 ( 4.11 - 4.88 ) | 4.42 ( 1384.77 ) | 4.4 ( 4.03 ) | 2.14 ( 2 ) |
| Somnolence | 435 | 4.42 ( 4.02 - 4.86 ) | 4.37 ( 1127.06 ) | 4.35 ( 3.95 ) | 2.12 ( 1.97 ) |
| Suicidal ideation | 361 | 6.14 ( 5.54 - 6.82 ) | 6.08 ( 1521.92 ) | 6.04 ( 5.44 ) | 2.59 ( 2.42 ) |
| Drug abuse | 347 | 4.9 ( 4.41 - 5.45 ) | 4.86 ( 1056.69 ) | 4.83 ( 4.34 ) | 2.27 ( 2.1 ) |
| Completed suicide | 345 | 4.58 ( 4.11 - 5.09 ) | 4.54 ( 946.28 ) | 4.51 ( 4.05 ) | 2.17 ( 2 ) |
| Insomnia | 341 | 2.27 ( 2.04 - 2.52 ) | 2.25 ( 237.77 ) | 2.25 ( 2.02 ) | 1.17 ( 1.01 ) |
| Intentional overdose | 329 | 6.79 ( 6.08 - 7.57 ) | 6.72 ( 1588.54 ) | 6.66 ( 5.97 ) | 2.74 ( 2.55 ) |
| Suicide attempt | 322 | 7.41 ( 6.64 - 8.28 ) | 7.35 ( 1747.6 ) | 7.27 ( 6.51 ) | 2.86 ( 2.67 ) |
| Confusional state | 309 | 4.23 ( 3.78 - 4.73 ) | 4.2 ( 748.86 ) | 4.17 ( 3.73 ) | 2.06 ( 1.88 ) |
| Overdose | 302 | 3.35 ( 2.99 - 3.76 ) | 3.33 ( 491.07 ) | 3.32 ( 2.96 ) | 1.73 ( 1.55 ) |
| Drug interaction | 290 | 3.55 ( 3.16 - 3.99 ) | 3.52 ( 522.94 ) | 3.51 ( 3.13 ) | 1.81 ( 1.63 ) |
| Agitation | 285 | 6.81 ( 6.06 - 7.66 ) | 6.76 ( 1385.32 ) | 6.7 ( 5.96 ) | 2.74 ( 2.54 ) |
| Tachycardia | 260 | 4.53 ( 4.01 - 5.12 ) | 4.5 ( 704.23 ) | 4.48 ( 3.96 ) | 2.16 ( 1.96 ) |
| Coma | 250 | 8.12 ( 7.16 - 9.2 ) | 8.06 ( 1528.55 ) | 7.97 ( 7.03 ) | 3 ( 2.77 ) |
| Anger | 246 | 12.33 ( 10.86 - 13.99 ) | 12.24 ( 2491.61 ) | 12.02 ( 10.59 ) | 3.59 ( 3.34 ) |
| Tremor | 236 | 2.69 ( 2.37 - 3.06 ) | 2.68 ( 247.61 ) | 2.67 ( 2.35 ) | 1.42 ( 1.22 ) |
| Aggression | 225 | 8.61 ( 7.55 - 9.83 ) | 8.56 ( 1482.34 ) | 8.45 ( 7.41 ) | 3.08 ( 2.84 ) |
| Restless legs syndrome | 189 | 19.82 ( 17.14 - 22.92 ) | 19.7 ( 3253.57 ) | 19.13 ( 16.54 ) | 4.26 ( 3.91 ) |
| Serotonin syndrome | 182 | 14.01 ( 12.09 - 16.24 ) | 13.93 ( 2138.24 ) | 13.65 ( 11.78 ) | 3.77 ( 3.46 ) |
| Intentional self-injury | 175 | 11.85 ( 10.2 - 13.77 ) | 11.79 ( 1696.85 ) | 11.59 ( 9.98 ) | 3.53 ( 3.23 ) |
| Withdrawal syndrome | 168 | 7.45 ( 6.39 - 8.67 ) | 7.41 ( 921.35 ) | 7.34 ( 6.3 ) | 2.87 ( 2.6 ) |
| Nightmare | 153 | 8.25 ( 7.03 - 9.68 ) | 8.21 ( 956.72 ) | 8.12 ( 6.92 ) | 3.02 ( 2.72 ) |
| Sopor | 144 | 14.86 ( 12.59 - 17.53 ) | 14.79 ( 1809.29 ) | 14.47 ( 12.26 ) | 3.86 ( 3.48 ) |
| Irritability | 141 | 3.99 ( 3.38 - 4.71 ) | 3.98 ( 312.63 ) | 3.96 ( 3.35 ) | 1.98 ( 1.71 ) |
| Electrocardiogram qt prolonged | 140 | 6.91 ( 5.85 - 8.17 ) | 6.89 ( 697.06 ) | 6.82 ( 5.77 ) | 2.77 ( 2.47 ) |
| Hallucination | 132 | 4.93 ( 4.15 - 5.85 ) | 4.91 ( 408.59 ) | 4.88 ( 4.11 ) | 2.29 ( 1.99 ) |
| Sleep disorder | 122 | 3.36 ( 2.81 - 4.01 ) | 3.35 ( 199.81 ) | 3.33 ( 2.79 ) | 1.74 ( 1.45 ) |
| Restlessness | 118 | 5.48 ( 4.57 - 6.57 ) | 5.46 ( 426.75 ) | 5.42 ( 4.52 ) | 2.44 ( 2.12 ) |
| Abnormal dreams | 115 | 6.85 ( 5.7 - 8.24 ) | 6.83 ( 566.43 ) | 6.77 ( 5.63 ) | 2.76 ( 2.42 ) |
| Panic attack | 110 | 4.22 ( 3.5 - 5.1 ) | 4.21 ( 267.84 ) | 4.19 ( 3.47 ) | 2.07 ( 1.75 ) |
| Disturbance in attention | 109 | 3.18 ( 2.64 - 3.85 ) | 3.18 ( 161.92 ) | 3.17 ( 2.62 ) | 1.66 ( 1.36 ) |
| Poisoning deliberate | 105 | 14.87 ( 12.25 - 18.05 ) | 14.82 ( 1322.31 ) | 14.5 ( 11.95 ) | 3.86 ( 3.4 ) |
| Hyponatraemia | 101 | 5.12 ( 4.21 - 6.23 ) | 5.1 ( 330.82 ) | 5.07 ( 4.17 ) | 2.34 ( 2 ) |
| Depressed level of consciousness | 97 | 4.35 ( 3.56 - 5.31 ) | 4.34 ( 247.66 ) | 4.32 ( 3.53 ) | 2.11 ( 1.77 ) |
| Tinnitus | 94 | 3.31 ( 2.7 - 4.05 ) | 3.3 ( 150.26 ) | 3.29 ( 2.69 ) | 1.72 ( 1.39 ) |
| Dysarthria | 94 | 4.11 ( 3.36 - 5.04 ) | 4.1 ( 219.38 ) | 4.08 ( 3.33 ) | 2.03 ( 1.69 ) |
| Disorientation | 93 | 4.4 ( 3.58 - 5.39 ) | 4.39 ( 241.59 ) | 4.36 ( 3.56 ) | 2.13 ( 1.78 ) |
| Mood swings | 88 | 4.21 ( 3.42 - 5.2 ) | 4.2 ( 213.56 ) | 4.18 ( 3.39 ) | 2.06 ( 1.71 ) |
| Abnormal weight gain | 87 | 26.12 ( 21.07 - 32.38 ) | 26.05 ( 2011.52 ) | 25.04 ( 20.2 ) | 4.65 ( 3.98 ) |
| Drug withdrawal syndrome | 86 | 2.81 ( 2.27 - 3.47 ) | 2.8 ( 99.44 ) | 2.8 ( 2.26 ) | 1.48 ( 1.14 ) |
| Depressed mood | 85 | 2.65 ( 2.14 - 3.28 ) | 2.64 ( 86.52 ) | 2.64 ( 2.13 ) | 1.4 ( 1.06 ) |
| Psychotic disorder | 83 | 4.3 ( 3.46 - 5.34 ) | 4.29 ( 208.1 ) | 4.27 ( 3.44 ) | 2.09 ( 1.72 ) |
| Neuroleptic malignant syndrome | 82 | 9.45 ( 7.6 - 11.76 ) | 9.43 ( 608.69 ) | 9.3 ( 7.48 ) | 3.22 ( 2.76 ) |
| Sedation | 81 | 5.61 ( 4.51 - 6.99 ) | 5.6 ( 303.61 ) | 5.56 ( 4.47 ) | 2.48 ( 2.08 ) |
| Rhabdomyolysis | 80 | 3.39 ( 2.72 - 4.22 ) | 3.38 ( 133.56 ) | 3.37 ( 2.7 ) | 1.75 ( 1.39 ) |
| Mania | 74 | 6.56 ( 5.22 - 8.25 ) | 6.55 ( 344.34 ) | 6.49 ( 5.16 ) | 2.7 ( 2.26 ) |
| Abnormal behaviour | 74 | 4.15 ( 3.3 - 5.21 ) | 4.14 ( 175.11 ) | 4.12 ( 3.28 ) | 2.04 ( 1.65 ) |
| Increased appetite | 73 | 8.1 ( 6.43 - 10.2 ) | 8.08 ( 447.33 ) | 7.99 ( 6.34 ) | 3 ( 2.53 ) |
| Paranoia | 70 | 5.91 ( 4.67 - 7.49 ) | 5.9 ( 282.51 ) | 5.86 ( 4.63 ) | 2.55 ( 2.11 ) |
| Dyskinesia | 69 | 3.76 ( 2.97 - 4.76 ) | 3.75 ( 138.57 ) | 3.74 ( 2.95 ) | 1.9 ( 1.5 ) |
