# Supplementary material for: Long-term safety evaluation of mirtazapine: A real-world pharmacovigilance study based on the FAERS database
Source: PLoS One. 2026 Mar 6;21(3):e0340092. doi: 10.1371/journal.pone.0340092 (PMC12965596; doi:10.1371/journal.pone.0340092)
Supplement: S5 Table — (DOCX) [file pone.0340092.s005.docx]

**Supplementary Table5**

**Top 50 positive AEs associated with mirtazapine at the PT level in patients aged ≥65 years**

| PT | Numbers | ROR(95%CI) | PRR(χ^2^) | EBGM(EBGM05) | IC(IC025) |
| --- | --- | --- | --- | --- | --- |
| Foetal exposure during pregnancy | 87 | 7.5 ( 6.04 - 9.31 ) | 7.18 ( 463.35 ) | 7.15 ( 5.76 ) | 2.84 ( 2.42 ) |
| Intentional overdose | 49 | 7.96 ( 5.98 - 10.58 ) | 7.77 ( 287.97 ) | 7.72 ( 5.81 ) | 2.95 ( 2.35 ) |
| Toxicity to various agents | 41 | 4.27 ( 3.13 - 5.82 ) | 4.2 ( 100.02 ) | 4.19 ( 3.07 ) | 2.07 ( 1.51 ) |
| Somnolence | 33 | 3.87 ( 2.74 - 5.46 ) | 3.82 ( 68.69 ) | 3.81 ( 2.7 ) | 1.93 ( 1.31 ) |
| Drug withdrawal syndrome neonatal | 24 | 14.6 ( 9.74 - 21.9 ) | 14.42 ( 296.27 ) | 14.25 ( 9.5 ) | 3.83 ( 2.63 ) |
| Intentional self-injury | 21 | 8.4 ( 5.45 - 12.93 ) | 8.31 ( 134.24 ) | 8.26 ( 5.36 ) | 3.05 ( 2.01 ) |
| Small for dates baby | 21 | 13.54 ( 8.78 - 20.86 ) | 13.39 ( 238.18 ) | 13.25 ( 8.59 ) | 3.73 ( 2.47 ) |
| Premature baby | 20 | 3.51 ( 2.25 - 5.45 ) | 3.48 ( 35.32 ) | 3.47 ( 2.23 ) | 1.8 ( 1 ) |
| Suicide attempt | 20 | 4.32 ( 2.78 - 6.71 ) | 4.28 ( 50.23 ) | 4.27 ( 2.74 ) | 2.09 ( 1.25 ) |
| Atrial septal defect | 20 | 9.36 ( 6.01 - 14.57 ) | 9.27 ( 146.52 ) | 9.2 ( 5.91 ) | 3.2 ( 2.09 ) |
| Tachycardia | 18 | 3.57 ( 2.24 - 5.69 ) | 3.55 ( 32.93 ) | 3.54 ( 2.22 ) | 1.82 ( 0.98 ) |
| Patent ductus arteriosus | 16 | 15.75 ( 9.6 - 25.85 ) | 15.62 ( 216.12 ) | 15.42 ( 9.4 ) | 3.95 ( 2.35 ) |
| Drug abuse | 14 | 5.6 ( 3.31 - 9.49 ) | 5.57 ( 52.29 ) | 5.55 ( 3.27 ) | 2.47 ( 1.34 ) |
| Maternal drugs affecting foetus | 14 | 9.97 ( 5.88 - 16.91 ) | 9.9 ( 111.17 ) | 9.83 ( 5.79 ) | 3.3 ( 1.88 ) |
| Anger | 13 | 4.69 ( 2.72 - 8.11 ) | 4.67 ( 37.36 ) | 4.65 ( 2.69 ) | 2.22 ( 1.11 ) |
| Serotonin syndrome | 13 | 16.69 ( 9.63 - 28.91 ) | 16.58 ( 187.66 ) | 16.36 ( 9.44 ) | 4.03 ( 2.18 ) |
| Hypospadias | 11 | 23.33 ( 12.82 - 42.46 ) | 23.2 ( 229.07 ) | 22.76 ( 12.51 ) | 4.51 ( 2.17 ) |
| Cryptorchism | 11 | 36.84 ( 20.17 - 67.26 ) | 36.62 ( 369.35 ) | 35.51 ( 19.45 ) | 5.15 ( 2.34 ) |
| Neonatal seizure | 11 | 42.52 ( 23.25 - 77.76 ) | 42.27 ( 427.51 ) | 40.8 ( 22.31 ) | 5.35 ( 2.39 ) |
| Exposure during pregnancy | 11 | 3.82 ( 2.11 - 6.92 ) | 3.81 ( 22.72 ) | 3.8 ( 2.1 ) | 1.93 ( 0.78 ) |
| Respiratory disorder neonatal | 11 | 14.09 ( 7.76 - 25.58 ) | 14.01 ( 131.36 ) | 13.85 ( 7.63 ) | 3.79 ( 1.9 ) |
| Miosis | 10 | 13.75 ( 7.36 - 25.7 ) | 13.68 ( 116.2 ) | 13.53 ( 7.24 ) | 3.76 ( 1.78 ) |
| Talipes | 8 | 13.92 ( 6.92 - 27.99 ) | 13.86 ( 94.34 ) | 13.71 ( 6.81 ) | 3.78 ( 1.53 ) |
| Lactic acidosis | 8 | 7.5 ( 3.74 - 15.06 ) | 7.47 ( 44.58 ) | 7.43 ( 3.7 ) | 2.89 ( 1.15 ) |
| Acute respiratory distress syndrome | 8 | 7.12 ( 3.55 - 14.3 ) | 7.1 ( 41.66 ) | 7.06 ( 3.52 ) | 2.82 ( 1.11 ) |
| Hyperreflexia | 7 | 19.58 ( 9.26 - 41.38 ) | 19.5 ( 120.85 ) | 19.19 ( 9.08 ) | 4.26 ( 1.52 ) |
| Poisoning | 7 | 13 ( 6.16 - 27.41 ) | 12.95 ( 76.35 ) | 12.82 ( 6.08 ) | 3.68 ( 1.34 ) |
| Renal aplasia | 7 | 60.5 ( 28.25 - 129.56 ) | 60.27 ( 387.63 ) | 57.31 ( 26.76 ) | 5.84 ( 1.78 ) |
| Tachypnoea | 7 | 5.43 ( 2.58 - 11.43 ) | 5.42 ( 25.1 ) | 5.4 ( 2.56 ) | 2.43 ( 0.77 ) |
| Mydriasis | 7 | 4.64 ( 2.21 - 9.76 ) | 4.63 ( 19.83 ) | 4.61 ( 2.19 ) | 2.21 ( 0.64 ) |
| Vitamin b1 decreased | 7 | 268.23 ( 117.67 - 611.47 ) | 267.2 ( 1505.21 ) | 216.83 ( 95.12 ) | 7.76 ( 1.83 ) |
| Psychomotor retardation | 7 | 40.43 ( 19 - 86.04 ) | 40.28 ( 259.05 ) | 38.95 ( 18.3 ) | 5.28 ( 1.72 ) |
| Withdrawal syndrome | 7 | 7.44 ( 3.53 - 15.67 ) | 7.42 ( 38.62 ) | 7.37 ( 3.5 ) | 2.88 ( 1.01 ) |
| Hyperlactacidaemia | 7 | 44.21 ( 20.75 - 94.19 ) | 44.04 ( 283.58 ) | 42.45 ( 19.92 ) | 5.41 ( 1.73 ) |
| Poor feeding infant | 7 | 9.86 ( 4.68 - 20.77 ) | 9.82 ( 55.03 ) | 9.75 ( 4.63 ) | 3.29 ( 1.19 ) |
| Ventricular septal defect | 7 | 5.7 ( 2.71 - 12 ) | 5.69 ( 26.91 ) | 5.66 ( 2.69 ) | 2.5 ( 0.81 ) |
| Clonus | 6 | 20.45 ( 9.11 - 45.91 ) | 20.39 ( 108.71 ) | 20.05 ( 8.93 ) | 4.33 ( 1.32 ) |
| Brain oedema | 6 | 5.57 ( 2.49 - 12.44 ) | 5.55 ( 22.31 ) | 5.53 ( 2.48 ) | 2.47 ( 0.65 ) |
| Syndactyly | 6 | 45.05 ( 19.9 - 101.99 ) | 44.91 ( 247.87 ) | 43.25 ( 19.11 ) | 5.43 ( 1.5 ) |
| Hypophagia | 6 | 5.41 ( 2.42 - 12.09 ) | 5.4 ( 21.41 ) | 5.38 ( 2.41 ) | 2.43 ( 0.63 ) |
| Cardiac output increased | 6 | 186.31 ( 78.54 - 441.98 ) | 185.7 ( 948.43 ) | 159.92 ( 67.41 ) | 7.32 ( 1.58 ) |
| Poisoning deliberate | 6 | 9.36 ( 4.19 - 20.94 ) | 9.34 ( 44.31 ) | 9.27 ( 4.14 ) | 3.21 ( 0.99 ) |
| Bradycardia neonatal | 6 | 12.28 ( 5.49 - 27.5 ) | 12.25 ( 61.33 ) | 12.13 ( 5.42 ) | 3.6 ( 1.13 ) |
| Agitation neonatal | 6 | 16.69 ( 7.44 - 37.41 ) | 16.64 ( 86.93 ) | 16.41 ( 7.32 ) | 4.04 ( 1.25 ) |
| Blood lactic acid decreased | 6 | 215.42 ( 89.97 - 515.84 ) | 214.71 ( 1074.79 ) | 180.97 ( 75.58 ) | 7.5 ( 1.57 ) |
| Congenital central nervous system anomaly | 6 | 44.76 ( 19.78 - 101.32 ) | 44.62 ( 246.25 ) | 42.98 ( 18.99 ) | 5.43 ( 1.5 ) |
| Electrocardiogram abnormal | 6 | 25.53 ( 11.35 - 57.41 ) | 25.45 ( 137.87 ) | 24.92 ( 11.08 ) | 4.64 ( 1.39 ) |
| Tremor neonatal | 5 | 40.72 ( 16.67 - 99.46 ) | 40.61 ( 186.56 ) | 39.25 ( 16.07 ) | 5.29 ( 1.21 ) |
| Infantile apnoea | 5 | 10.29 ( 4.26 - 24.84 ) | 10.26 ( 41.43 ) | 10.18 ( 4.21 ) | 3.35 ( 0.82 ) |
| Plagiocephaly | 5 | 43.83 ( 17.92 - 107.18 ) | 43.71 ( 200.99 ) | 42.14 ( 17.23 ) | 5.4 ( 1.22 ) |
